# A Novel Prophage-like Insertion Element within *yabG* Triggers Early Entry into Sporulation in *Clostridium botulinum*

**DOI:** 10.3390/v15122431

**Published:** 2023-12-14

**Authors:** François P. Douillard, Inês Martins Portinha, Yağmur Derman, Cédric Woudstra, Tommi Mäklin, Martin B. Dorner, Hannu Korkeala, Adriano O. Henriques, Miia Lindström

**Affiliations:** 1 Department of Food Hygiene and Environmental Health, Faculty of Veterinary Medicine, University of Helsinki, 00350 Helsinki, Finland; francois.douillard@helsinki.fi (F.P.D.); ines.portinha@helsinki.fi (I.M.P.); yagmur.derman@helsinki.fi (Y.D.); cedric.woudstra@sund.ku.dk (C.W.); hannu.korkeala@helsinki.fi (H.K.); 2 Department of Mathematics and Statistics, Faculty of Science, University of Helsinki, 00560 Helsinki, Finland; tommi.maklin@helsinki.fi; 3 Centre for Biological Threats and Special Pathogens, ZBS3—Biological Toxins, Robert Koch Institute, 13353 Berlin, Germany; dornerm@rki.de; 4 Institute of Chemical and Biological Technology, NOVA University Lisbon, 2780-157 Oeiras, Portugal; aoh@itqb.unl.pt

**Keywords:** *Clostridium botulinum*, mobile element, sporulation, prophage, *yabG*

## Abstract

Sporulation is a finely regulated morphogenetic program important in the ecology and epidemiology of *Clostridium botulinum*. Exogenous elements disrupting sporulation-associated genes contribute to sporulation regulation and introduce diversity in the generally conserved sporulation programs of endospore formers. We identified a novel prophage-like DNA segment, termed the *yin* element, inserted within *yabG*, encoding a sporulation-specific cysteine protease, in an environmental isolate of *C. botulinum*. Bioinformatic analysis revealed that the genetic structure of the *yin* element resembles previously reported mobile intervening elements associated with sporulation genes. Within a pure *C. botulinum* culture, we observed two subpopulations of cells with the *yin* element either integrated into the *yabG* locus or excised as a circular DNA molecule. The dynamics between the two observed conformations of the *yin* element was growth-phase dependent and likely mediated by recombination events. The *yin* element was not required for sporulation by *C. botulinum* but triggered an earlier entry into sporulation than in a related isolate lacking this element. So far, the *yin* element has not been found in any other *C. botulinum* strains or other endospore-forming species. It remains to be demonstrated what kind of competitive edge it provides for *C. botulinum* survival and persistence.

## 1. Introduction

Some bacteria escape starvation and other adverse conditions by forming resistant endospores through a time- and energy-consuming differentiation process called sporulation, elaborately described in several *Bacillus*, *Clostridium*, and related species [1,2,3,4]. The process is coordinated and timely controlled by multiple sigma factors and accessory transcriptional regulators. Spores can survive extreme conditions over long periods of time [5,6] and, for pathogenic species, play a major role in the epidemiology of bacterial diseases, such as anthrax caused by *Bacillus anthracis* [7], *Clostridioides difficile* infection [8], tetanus caused by *Clostridium tetani* [9], *Bacillus cereus* infection [10], and botulism caused by *Clostridium botulinum* [11]. Environmental spores of *C. botulinum* are of major concern for the food industry due to the risk of food-borne botulism [12] and can also pose direct risks to humans through exposure and colonization of the gut, causing toxicoinfectious botulism in infants [13] or in at-risk adults [14,15].

Mobile genetic elements can contribute to the regulation of sporulation. In some of the *B. subtilis* and *C. difficile* strains, gene encoding the late-stage mother-cell sigma-factor SigK (σ^K^) is disrupted by an intervening prophage-like element, termed the *skin* element. This element is timely excised during sporulation, resulting in a functional *sigK* to let sporulation proceed [16,17,18]. Other sporulation-related genes interrupted by intervening prophage-like elements include *spsM* of *B. subtilis*, required for the maturation of the spore surface layers and interrupted by the SPβ prophage [19]; *spoVFB* of *Bacillus weihenstephanensis* coding for the β-subunit of dipicolinic acid synthase and interrupted by the *vfbin* element [20]; and *gerE* of *Bacillus cereus* coding for a transcription factor that works with SigK to control the late stages of the mother cell of gene expression [21]. These reports underline a prevalence for mobile genetic elements (prophage) inserted in sporulation-associated genes and suggest direct or indirect modulation of the sporulation cascade, perhaps providing an ecological benefit to the host bacteria. Such bacteria-phage interactions relate to active lysogeny, where the prophage disrupts the target gene expression and its regulation and therefore acts as a regulatory switch (phage-RS) [22].

The *yabG* is regulated by SigK [23] and codes for a cysteine protease that regulates the assembly of the spore coat by processing several coat proteins in *B. subtilis* [24,25]. YabG also processes two proteins required for spore germination in *C. difficile*, CspBA and Pre-pro-SleC, as well as a coat protein required for efficient host colonization [26,27]. Interestingly, *yabG* is the only member of the SigK regulon that is conserved across spore formers and part of a genomic signature for sporulation [28]. Of note, SigK is known as a late sporulation factor but has also shown an early role in sporulation across several clostridial species [5]. In our previous work [29], we sequenced the genome of a *C. botulinum* strain (V73) isolated from household dust and related to a case of infant botulism. We identified a prophage-like sequence disrupting *yabG* (hereinafter referred to as the *yin* element, for the *yabG*-intervening element). To test the hypothesis that the *yin* element has a role in the control of sporulation in *C. botulinum*, we first compared the phenotypes of V73 and its counterpart devoid of the *yin* element (ST7B). We showed that the *yin* element was temporally excised from the chromosome and triggered an earlier entry into sporulation in V73 compared with ST7B. This work describes, for the first time, a regulatory prophage-like insertion element within *yabG* and further illustrates the diversity encountered in the regulation of the sporulation program in endospore formers while raising questions on the ecological benefit of such an intervening element for persistence and survival of the bacteria.

## 2. Materials and Methods

### 2.1. Bacterial Growth and DNA Isolation

*C. botulinum* isolates V73 and ST7B are associated with an infant botulism case and share a recent common ancestor [29,30]. Both strains were cultured anaerobically at 37 °C overnight in trypticase–peptone–glucose–yeast-extract (TPGY) broth, and genomic DNA was extracted using Wizard^®^ Genomic DNA Purification Kit (Promega, Madison, WI, USA), as per the manufacturer’s instructions. The DNA concentration was determined using the Qubit fluorometric assay (Thermo Scientific, Waltham, MA, USA).

### 2.2. Genome Sequencing and Bioinformatic Analysis

The genome of *C. botulinum* V73 was sequenced using the PacBio sequencer (Pacific Biosciences, Menlo Park, CA, USA). PacBio library preparation, genome assembly, and polishing were carried out as previously described [29]. The complete and closed genome of V73 was obtained using both PacBio sequencing reads (this work) and Illumina paired-end reads (SRR11288493) [29]. Genome assembly of *C. botulinum* V73 was analyzed using the Microbial Genomes Atlas (MiGA) web interface [31]. Prophages were identified using the Phaster online analysis tool [32]. The genome of V73 was annotated using various tools, including PATRIC [33], RASTtk [34], and Prokka [35]. Gene annotation of the *yin* element was also conducted using the Phyre2 web portal [36]. Protein-structure prediction and models of the complexes between recombination directionality factors (RDFs) and their associated recombinases were generated using AlphaFold [37]. AlphaFold2 was used for structural modeling of individual proteins and complexes [37]. We first obtained models for SpoIVCA, SprA (*B. subtilis*), and CD1231 (*C. difficile*) recombinases and their cognate directionality factors SkrB, SprB, and CD1234 and for the *yin*-encoded recombinase (HEQ52_18460). The models were obtained by uploading the sequences to the open access AlphaFold2 Colab notebook (https://colab.research.google.com/github/deepmind/alphafold/blob/main/notebooks/AlphaFold.ipynb, accessed on 16 August 2022) [38]. We also generated models for putative complexes between the recombinases and their known or predicted directionality factors (as above). The interaction was predicted to involve the C-terminal end of the recombinases (Figure S1). We then generated models between the *yin* recombinase (HEQ52_18460) and the predicted *yin*-encoded proteins with 250 amino acid residues or fewer. Only one of the selected *yin*-encoded proteins, HEQ52_18460 (Table S1), was predicted to form a complex with the HEQ52_18485 recombinase. The interaction was predicted to form at the C-terminal region of the recombinase [39]. Five models were generated for each protein or protein complex. The confidence of the modeling was assessed by the pLDDT metric and the predicted alignment error (PAE), i.e., uncertainty about the interface. Values of pLDDT > 90 were expected to be highly accurate. Structural representations were generated using PyMOL Molecular Graphics System (Schrödinger, Inc., New York, NY, USA).

### 2.3. Growth Curves

The growth of *C. botulinum* ST7B and V73 was monitored using the Hidex Sense multi-well plate reader (Hidex Oy, Turku, Finland). Briefly, following two consecutive overnight sub-cultures in TPGY medium at 37 °C in anaerobic conditions, cultures of the two isolates were diluted 1:100 in 5 mL of TPGY broth. Then, 200 μL of the resulting suspension were added per well of a 96-well plate. The plate was then placed into the Hidex Sense plate reader and incubated at 37 °C anaerobically. Optical density at 600 nm was recorded every 15 min for 24 h. The results were based on three biological replicate series consisting of four technical replicates of each isolate.

### 2.4. Toxin Quantification

*C. botulinum* V73 and ST7B cultures were grown at 37 °C anaerobically in TPGY medium. Each strain was inoculated 1:100 into 5 mL of fresh TPGY medium. Samples were collected for up to 24 h and stored at −80 °C. Botulinum neurotoxin (BoNT) type A1 was quantified using a sandwich-ELISA, as previously described [40,41], in triplicate.

### 2.5. Spore Count Assay and Spore Heat Resistance

*C. botulinum* V73 and ST7B cultures were grown anaerobically at 37 °C. Total viable cell and heat-resistant spore counts were measured at 48, 72, and 168 h after inoculation, as previously described [42]. Spore heat resistance of *C. botulinum* V73 and ST7B was studied as described in [43], by heating for variable times at 98 °C and counting the survivors.

### 2.6. Phase-Contrast Microscopy

Phase-contrast microscopy was used to monitor *C. botulinum* V73 and ST7B cultures at 5, 24, 48, 72, 96, 120, and 240 h after inoculation. Sample preparation, microscopic observation, and cell counts were performed as previously described [43].

### 2.7. Spore Germination Assay

Spores of *C. botulinum* ST7B and V73 were prepared as previously described [43]. The spores were then heat-activated at 80 °C for 15 min and OD-adjusted to ~0.5 in Tris-HCl (20 mM, pH 7.4) buffer with 50 mM of NaHCO_3_, 50 mM of sodium L-lactate, and either L-alanine (50 mM) or L-cysteine (50 mM). The microtiter plate containing the samples was then placed into the Hidex Sense multi-well plate reader and incubated anaerobically at 37 °C under double orbital shaking. Optical density at 600 nm was recorded every 5 min for 24 h. Three technical replicates per strain per condition were included.

### 2.8. Transmission Electron Microscopy (TEM) Analysis

*C. botulinum* V73 and ST7B spore samples were purified and prepared for thin sectioning TEM analysis [43]. The grids were visualized using a JEM-1400 transmission electron microscope (JEOL Ltd., Tokyo, Japan) at the Electron Microscopy Unit (EMBI, Institute of Biotechnology, University of Helsinki).

### 2.9. Detection and Quantification of the Different Forms of yin

From an overnight culture, *C. botulinum* ST7B and V73 were inoculated at a 1:100 dilution in TPGY medium and grown anaerobically at 37 °C. After 5, 24, 48, 72, 96, and 120 h of growth, genomic DNA from 1 mL of culture was extracted as described above. Segments of the *yin* element (amplicon spanning across *yabG* and the *yin* element), the intact *yabG* coding sequence (internal amplicon), and the circular *yin* element were amplified by PCR using HotStarTaq Master Mix Kit (Qiagen GmbH, Hilden, Germany) (Table S1) and visualized by agarose gel electrophoresis to detect the different *yin* element conformations over culture time. Finally, some of these amplicons were sent for Sanger sequencing (Macrogen Europe B.V., Amsterdam, The Netherlands). Using quantitative PCR and Maxima SYBR Green qPCR Master Mix (Thermo Scientific, Waltham, MA, USA), we further quantified the different *yin* element conformations at all time points. We amplified and quantified one fragment of *yabG* spanning across the whole *yin* element (intact *yabG* and excised *yin*) and one fragment spanning across one of the *att* sites (chromosomally integrated *yin*). As a reference, we amplified one segment of *yabG* that is not altered by the excision of the *yin* element.

### 2.10. Genome Sequence Accession Numbers

The closed genome of *C. botulinum* V73 was deposited in NCBI under the accession numbers CP050820 (chromosome) and CP050821 (plasmid).

## 3. Results and Discussion

### 3.1. Identification of a Prophage-like Intervening Element in yabG

**Figure 1:**
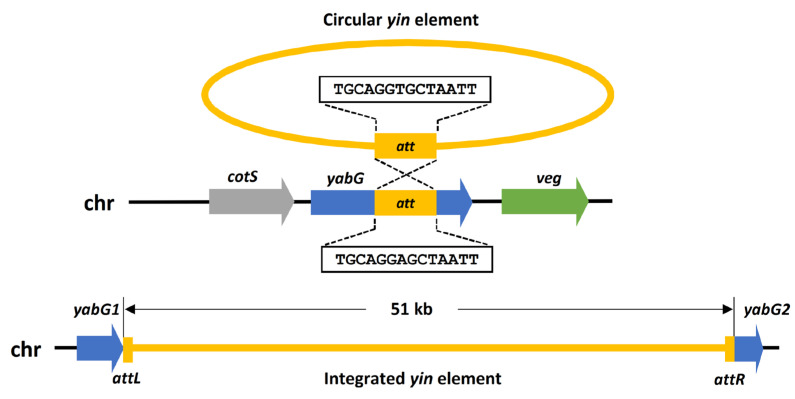
Schematic overview of the *yabG*-inserting (*yin*) element identified in *C. botulinum* strain V73. Legend: chr, chromosome; *att*, attachment sites; *attP*, for prophage; *attB*, for bacterial chromosome; *attL* and *attR*, left and right sites resulting from integration.

We recently showed that *C. botulinum* ST7B (genome accession numbers: CP050251, CP050252) and V73 are genetically related and essentially differ by the presence of one prophage based on read mapping using Illumina next-generation sequencing. Beside the presence of the *yin* element in *C. botulinum* V73, there were five SNPs/InDels different between the ST7B and V73 genomes: two located in intergenic regions and three within two genes (encoding a MurR/RpiR family transcriptional regulator or a histidine kinase) [29]. These SNPs/InDels are unlikely to impact the sporulation phenotype of *C. botulinum* ST7B and V73 [29]. To confirm the previously published Illumina next-generation sequencing results, we closed and annotated the genome of *C. botulinum* V73. The genome assembly confirmed the presence of a prophage located within *yabG* (Figure 1). *yabG* encodes a sporulation-specific protease shown to regulate the assembly of the spore coat in both *B. subtilis* and *C. difficile* [23,24,25,27].

The *yin* element was indeed predicted to be a putatively functional prophage and is genetically related to the class *Caudoviricetes* associated with *Clostridium* hosts (NC_029001, NC_007917, NC_028996, NC_028991, and NC_029004) or *Brevibacillus* hosts (NC_029029). Some of the predicted genes present in insertional conjugative elements (ICE) or prophage-mediated DNA rearrangement elements of endospore formers [17,18,19] can also be found in the *yin* element, as further detailed in Table S2. Within the *yin* element, the first open reading frame (HEQ52_18485) located downstream of the 5′-end of *yabG* encodes a putative recombinase (integrase) with significant Pfam-A matches to the resolvase (PF00239, E-value = 1.0 × 10^−38^), recombinase (PF07508, E-value = 5.8 × 10^−22^), and recombinase zinc-beta-ribbon-domain protein families (PF13408, E-value = 4.9 × 10^−13^) (Pfam 35.0 database [44]). The recombinase HEQ52_18485 shares 27% amino acid identity (100 out of 372 residues) with the SpoIVCA recombinase present in *skin* of *B. subtilis* [16] and 25% amino acid identity (87/352) with the resolvase CD630_12340 associated with sporulation and excision of *skin* in *C. difficile* [17]. Both *spoIVCA* in *B. subtilis* and HEQ52_18485 of *C. botulinum* V73 are located downstream of the 5′-end region of their respective disrupted gene (*sigK* and *yabG*) and in a similar orientation.

In addition to the recombinase, intervening elements typically require a recombination directionality factor (RDF) [18]. Using AlphaFold, we identified a putative RDF encoded within the *yin* element (Figure S1). When compared with the *skin* element of *C. difficile*, the putative RDF HEQ52_18460 differed from CD1234 in size (72 amino acids vs. 53 amino acids) and pI (5.5 vs. 4.9). Yet, based on AlphaFold protein-structure prediction and models of the complexes between the recombinase and the RDF of different intervening elements, i.e., SpoIVCA and Skr, CD1231 and CD1234, or SprA and SprB, the RDFs were all different, but models predicted that they interact with the same region of their associated recombinase (Figure S1). We also possibly identified a putative SigK-dependent promoter upstream of HEQ52_18460 (Figure S2), suggesting its regulation by the late-sporulation sigma-factor SigK. This is in line with the expression of *yabG* being under the control of SigK [23]. Excision of the *yin* element would reconstitute *yabG* under SigK control. The RDF candidate (HEQ52_18460) identified in the *yin* element remains to be further studied experimentally. To the best of our knowledge, the *yin* element is unique: None of the 26 isogenic *C. botulinum* isolates collected over time from the single infant botulism case [29], nor any available *C. botulinum* or other bacterial genomes deposited in NCBI, harbored the *yin* element (as of November 2023). Moreover, no other insertion elements were found to disrupt *yabG* in the genomes of spore-forming bacteria in public databases.

### 3.2. Phenotypic Impact of the yin Element on Sporulation and Toxin Production

**Figure 2:**
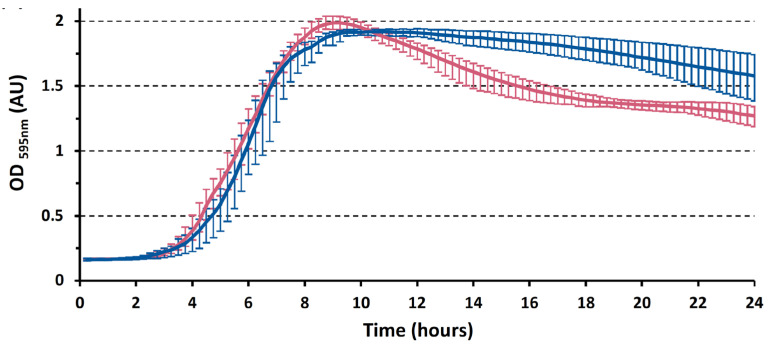
Growth curves of *C. botulinum* ST7B (blue) and V73 (pink). The experiment consisted of three biological replicates, each made of four technical replicates. Error bars represent minimum and maximum values among technical replicates.

The *yin* element is located within a sporulation gene in *C. botulinum* V73. Based on previously described prophage elements regulating gene expression, we considered the possibility that the *yin* element could play a similar regulatory role on *yabG* expression through timed self-excision. We investigated the impact of the *yin* element on the phenotype of *C. botulinum* V73 against its *yin*-devoid counterpart *C. botulinum* ST7B. *C. botulinum* V73 and ST7B cultures showed similar growth until entry into the stationary phase (Figure 2), suggesting that the *yin* element does not impact the overall physiology of the vegetative cells or cultures.

However, the growth curves of V73 and ST7B showed distinct profiles in the stationary phase, where a prominent drop in the optical density of the V73 culture likely reflected a higher number of cells entering sporulation compared with that of ST7B (Figure 2).

**Figure 3:**
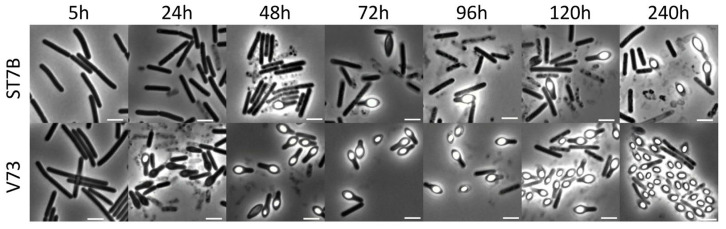
Phase-contrast microscopy observations of *C. botulinum* ST7B and V73 cultures in TPGY up to 240 h. Scale bar shown in each picture is 1 µm.

**Figure 4:**
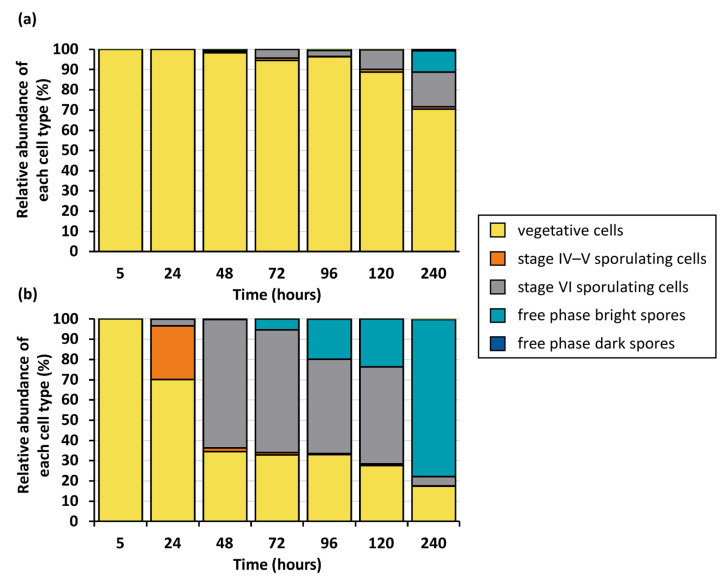
Relative abundance of different cell types in *C. botulinum* ST7B (**a**) and V73 (**b**) over time-based phase-contrast microscopy counting. Detailed counts can be found in Supplementary Materials (Table S3).

Phase-contrast microscopy of the *C. botulinum* V73 and ST7B cultures showed that the V73 culture initiated sporulation earlier and yielded higher free-spore counts than the ST7B culture (Figure 3, Figure 4, and Figure S3). Accordingly, after 240 h, the V73 culture consisted mostly of free spores, whereas in the ST7B culture, several cell types were still represented (Figure 3 and Figure 4). Thus, the distinct growth profiles of ST7B and V73 in stationary phases were likely due to different numbers of cells undergoing sporulation (Figure 2). In regard to the earlier entry into sporulation by *C. botulinum* V73 compared with ST7B, possible expression control of *yabG* by SigK and by other regulators remains to be further explored and understood, especially since SigK also showed an early role in sporulation across several clostridial species [5].

The sporulation master regulator Spo0A controls BoNT production in *C. botulinum* Group II type E [45]. Due to the apparent link between sporulation and toxinogenesis in *C. botulinum* Group II, we examined whether the presence of the *yin* element, through impacting sporulation, may also result in distinct levels of BoNT production between ST7B and V73. Based on ELISA quantification, the V73 culture produced a slightly higher level of botulinum neurotoxin (BoNT) than ST7B after 24 h (Figure S4). However, the difference was marginal and may be explained by the faster release of BoNT through efficient sporulation (Figure 3 and Figure 4) in the V73 cultures compared with ST7B cultures. Hence, the *yin* element is not likely to play a direct role in BoNT production or its regulation in the conditions tested.

### 3.3. Restoration of Intact yabG by Chromosomal Excision of the yin Element upon Sporulation

*C. botulinum* V73 and ST7B are phylogenetically related and essentially differ by the presence of the *yin* element. Sequence analysis of the *yabG* region of ST7B led us to identify a 14-bp repeat sequence flanking an inverted repeat sequence present at both extremities of the *yin* element (*att*R and *att*L sites), constituting the putative recombination sites recognized by the predicted site-specific recombinase HEQ52_18485 (Figure 1 and Table S2). Work performed on the *skin* element [17] and the presence of putative attachment sites (*att*) and site-specific recombinase within the *yin* element led us to hypothesize that the *yin* element of V73 is excised, yielding a restored *yabG* and a circularized *yin* element. To test this possibility, we designed sets of primers that aimed to amplify (i) a chromosomally integrated *yin* element, (ii) an intact *yabG* gene, and (iii) an excised and circularized *yin* element (Figure 1) and monitored their accumulation along time during sporulation.

**Figure 5:**
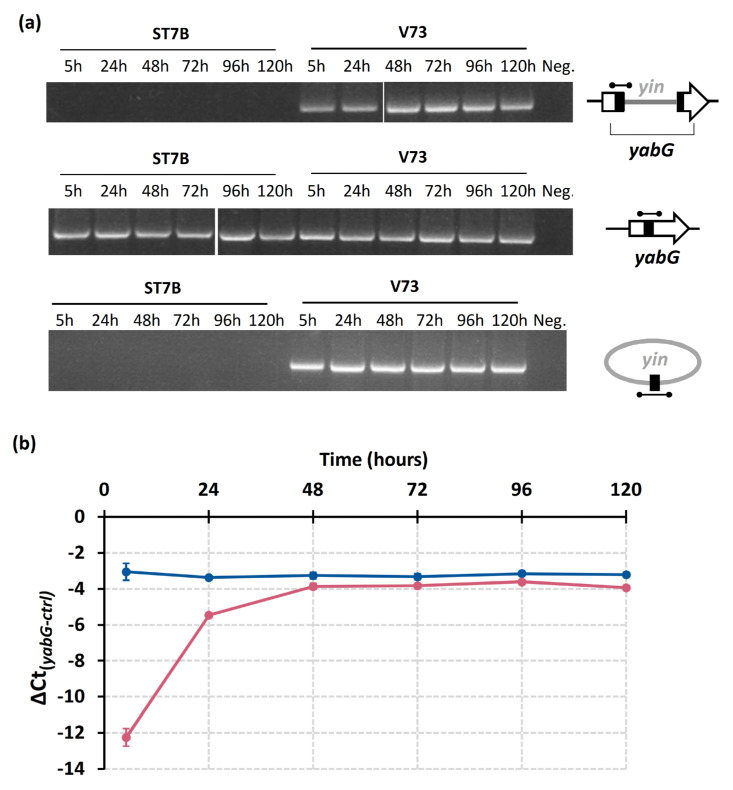
Plasticity of the *yin* element over time. (**a**) PCR amplification of the *yin* element integrated into the chromosome, the intact *yabG* gene, and the circular *yin* element in *C. botulinum* ST7B and V73 grown in TPGY medium. DNA-free water was used as a negative control. The amplicons (black bars) are shown near each DNA gel. Legend: Neg., negative control. (**b**) Quantitative PCR results to determine the relative proportion of restored *yabG* gene in *C. botulinum* ST7B and V73 over time. *yabG* primers span across the *att* recombination sites, whereas the control primers amplify a region elsewhere in the *yabG* gene. Error bars represent the standard deviations based on four technical replicates. Legend: pink curve, strain V73; blue curve, strain ST7B.

The *yin* element was found chromosomally integrated at all time points in V73 (Figure 5a). Moreover, amplicons corresponding to intact *yabG* were also observed at any time points in V73 (Figure 5a), suggesting that *yabG* was reconstituted through chromosomal excision of the *yin* element at all time points. In line with detecting the intact *yabG*, we also detected circularized *yin* (Figure 5a, further discussed below). The observations suggest a heterogeneous population with cells carrying a *yin* element either integrated in *yabG* or excised to produce a circular DNA element. Temporal excision of the *skin* element during sporulation was reported in *C. difficile* [17,18] and made us hypothesize that the dynamics of the *yin* element between chromosomal integration and excision into circular DNA could be temporally regulated. Indeed, the *C. botulinum* V73 population harbored a smaller proportion of cells with a restored *yabG* at 5 and 24 h than at later times, suggesting that an increasing proportion of bacterial cells harbored an intact *yabG* upon initiation of sporulation (Figure 5b). Upon excision of the *yin* element, spliced *yabG* was restored as an intact *yabG* gene in V73 (Figure S5). The restored *yabG* retained the 14-bp repeat (*att*B) required for recombination, as in ST7B (Figure S6). The *att*B is also present and identical to the sequence of *yabG* in other *C. botulinum* strains, such as CDC_297, A634, and CDC_69096, suggesting that the *yin* element could integrate into the chromosome at the *yabG* locus in other *C. botulinum* strains.

### 3.4. Circularization of the yin Element upon Chromosomal Excision

Recombinases mediate the excision and circularization of the *skin* element in *B. subtilis* and *C. difficile* [16,17,46,47]. To verify whether the *yin* element became circular upon excision from the chromosome, we designed primers located at both extremities of the *yin* element and in divergent orientation. Therefore, PCR products could only be generated upon amplification from the predicted circular *yin* element. Amplicons were obtained at all time points tested (Figure 5a), indicating that the excised *yin* element was indeed circular. This is in line with the aforementioned observations that an intact *yabG* was reconstituted at all time points tested. Sequencing of these amplicons revealed the *att*P with the same 14-bp repeat sequence flanked by asymmetric overlapping sequences (Figure S7), confirming that the recombination events are indeed mediated through *att* sites.

### 3.5. Presence of the yin Element Does Not Affect Spore Heat Resistance

**Figure 6:**
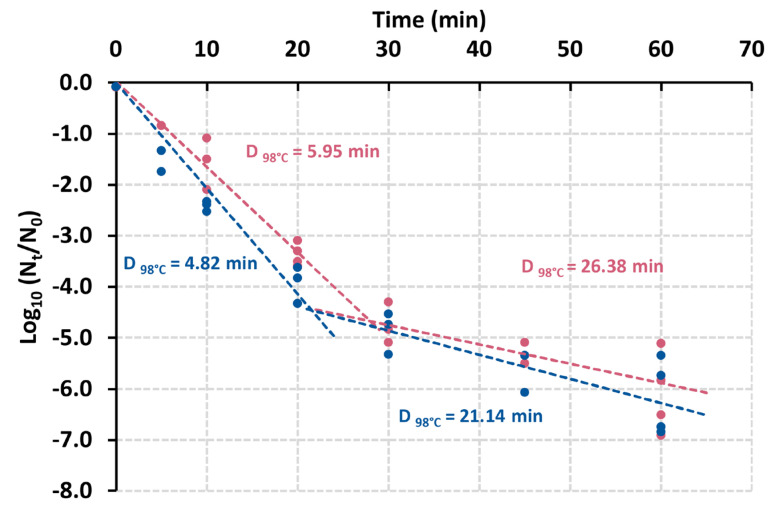
Thermal destruction of *C. botulinum* ST7B spores (pink dots) and V73 spores (blue dots). Purified spore suspensions were adjusted at OD_600nm_ 1 and heat-treated at 98 °C for different incubation times. D-values were obtained from the linear regression lines for each dataset. We observed the presence of two spore subpopulations, as shown with the two distinct destruction curves fitted on the data of each strain. The heat resistance of V73 spores appeared generally slightly lower than that of ST7B spores; however, the difference was statistically significant (*p* < 0.05, *t*-test) only for the most heat-sensitive spore subpopulations depicted during the first 20 min of the experiment.

Since YabG is involved in the assembly of the spore surface in *B. subtilis* [24,25] and spore germination in *C. difficile* [26,27], we hypothesized that the presence/absence of the *yin* element could control the function of YabG and therefore alter the spore properties. Thermal destruction assays showed that the V73 spores were slightly less heat resistant than spores of ST7B (most heat-sensitive spore subpopulations, depicted in Figure 6 in the first 20 min of heating); however, the difference was not statistically significant for the most heat-resistant spore subpopulations (depicted in Figure 6 after the first 20 min of heating).

Overall, the presence of the *yin* element had therefore no significant impact on the spore heat-resistance properties of V73 and ST7B. In line with this, transmission electron microscopy of purified V73 and ST7B spore preparations did not reveal any obvious structural differences between ST7B and V73 spores (Figure S8). Moreover, the germination patterns of V73 and ST7B spores were comparable (Figure S9). Further comparative proteomic analysis and biochemical characterization of the spores may bring further insights into the role of the *yin* element and *yabG* in *C. botulinum* sporulation.

## 4. Conclusions

We report the presence of a unique intervening mobile genetic element within *yabG*. This *yin* element is a prophage and has a unique gene content and order. *yabG* is conserved among spore-formers and is important for sporulation in at least *B. subtilis* and *C. difficile*; the *yin* element thus conforms to the general pattern that phage-like elements, such as the *skin* element and others, insert in sporulation-associated genes. It shares a number of common features with other intervening elements described in endospore formers: (i) prophage-like sequence, (ii) localization within a gene involved in sporulation, (iii) circularization of the element upon excision from the chromosome, (iv) temporal control of excision yet heterogeneous at population level, and (v) sporulation modulation. The *yin* element is unique to *C. botulinum* V73, as opposed to other intervening elements that can be found in multiple species or strains. Yet, the *attB* site present within *yabG* is conserved in other strains, suggesting that the *yin* element could be transferred among strains and integrated into the chromosome of other strains. It is likely that the *yin* element, like other intervening elements, originated from a temperate phage and with temporal patterns but may not yield to lytic production, as proposed in other intervening elements and phage-RS [19,22]. We detected excised *yin* elements in our cultures at all time points, suggesting heterogeneity in the population. We observed an earlier entry into sporulation and a more efficient production of free spores based on microscopy cell count analysis in *C. botulinum* V73 cultures than in its *yin*-devoid counterpart ST7B, indicating that *C. botulinum* ST7B may be more adapted to growth-supporting conditions than *C. botulinum* V73. Yet, it remains to be understood under which environmental conditions or ecological niches the *yin* element is beneficial to the host bacteria.

## Data Availability

The closed genome of *C. botulinum* V73 was deposited in NCBI under the accession numbers CP050820 (chromosome) and CP050821 (plasmid).

## Supplementary Materials

The following supporting information can be downloaded at https://www.mdpi.com/article/10.3390/v15122431/s1: Figure S1: AlphaFold protein-structure prediction and models of the complexes between the recombinase and its associated recombination directionality factors; Figure S2: Identification of a putative SigK-dependent promoter upstream of HEQ52_18460 in *C. botulinum* V73; Figure S3: Viable cell count and spore count of *C. botulinum* ST7B and V73; Figure S4: BoNT production of *C. botulinum* ST7B and V73 in TPGY medium; Figure S5: Sanger sequencing of the restored *yabG* gene in V73 at different time points; Figure S6: Identification of the attachment sites (*att*) based on the genome sequence of *C. botulinum* V73 and ST7B; Figure S7: Sanger sequencing of the *attP* site present in the circular *yin* element in *C. botulinum* V73; Figure S8: Transmission-electron-microscopy observations of individual *C. botulinum* ST7B and V73 spores; Figure S9: Spore germination assays of *C. botulinum* ST7B and V73. Table S1: Primers used in the present study; Table S2: List of genomic features predicted in the *yin* element; Table S3: Raw microscopy cell counts.
