# Supplementary material for: A Novel Prophage-like Insertion Element within yabG Triggers Early Entry into Sporulation in Clostridium botulinum
Source: Viruses. 2023 Dec 14;15(12):2431. doi: 10.3390/v15122431 (PMC10747680; doi:10.3390/v15122431)
Supplement: Supplementary file 1 [file viruses-15-02431-s001.zip › viruses-2442791-supplementary.pdf]

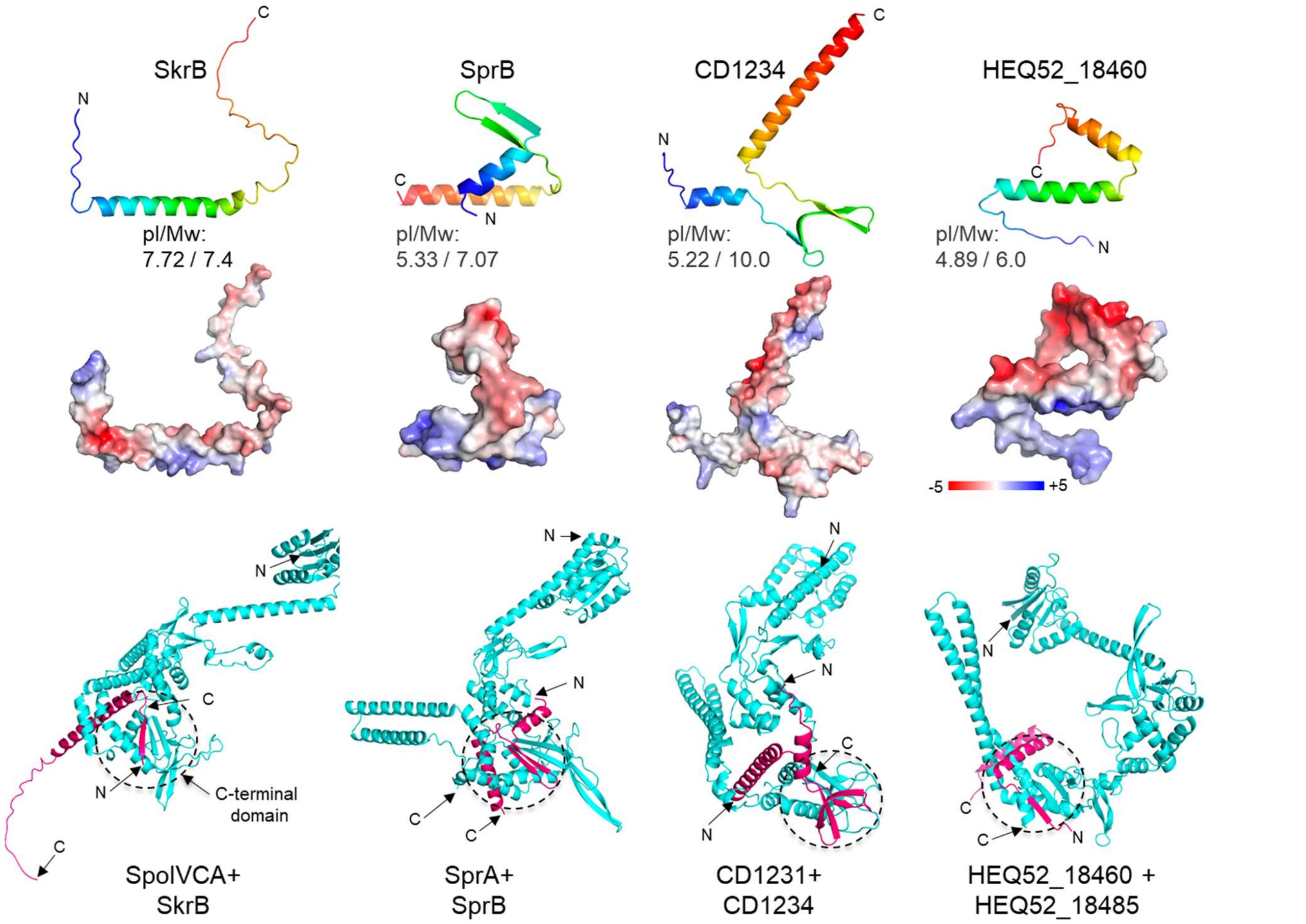

Figure S1. AlphaFold protein structure prediction and models of the complexes between the recombinase and its associated recombination directionality factor (RDF). The figure shows structural models generated by AlphaFold2 of the directionality factors SkrB, SprB, CD1234 and the putative *C. botulinum* factor HEQ52_18460 in cartoon representation colored from the N- (blue) to the C-terminus (red) (top row). The middle row shows the surface potential of the proteins. The bottom row shows the predicted complexes formed by the indicated recombinases (cyan) and their cognate recombination directionality factors (RDFs; dark pink), including the complex between the HEQ52_18485 recombinase and the putative factor HEQ52_18460. The C-terminal domain of the recombinases is indicated by a dashed circle. The N- and C-termini of the various proteins are indicated. Note that although the RDFs vary in size, structural elements and surface potential, they all interact with the C-terminal domain of the recombinases. Only the best (rank 1) AlphaFold2-generated models are shown.

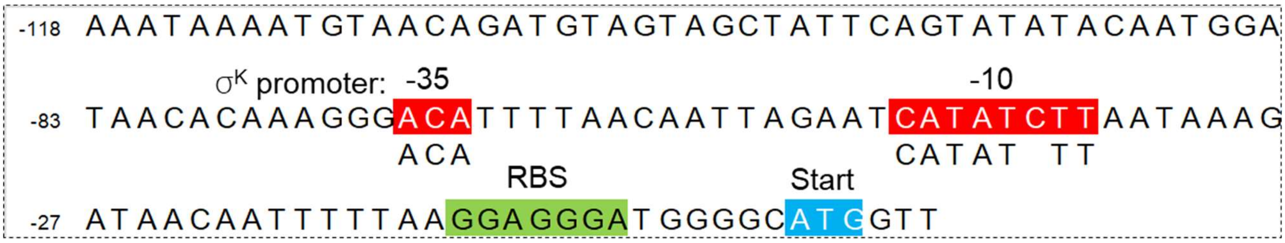

Figure S2. Identification of a putative SigK-dependent promoter upstream of the gene coding for the putative recombination directionality factor (RDF) HEQ52_18460 in *C. botulinum* V73.

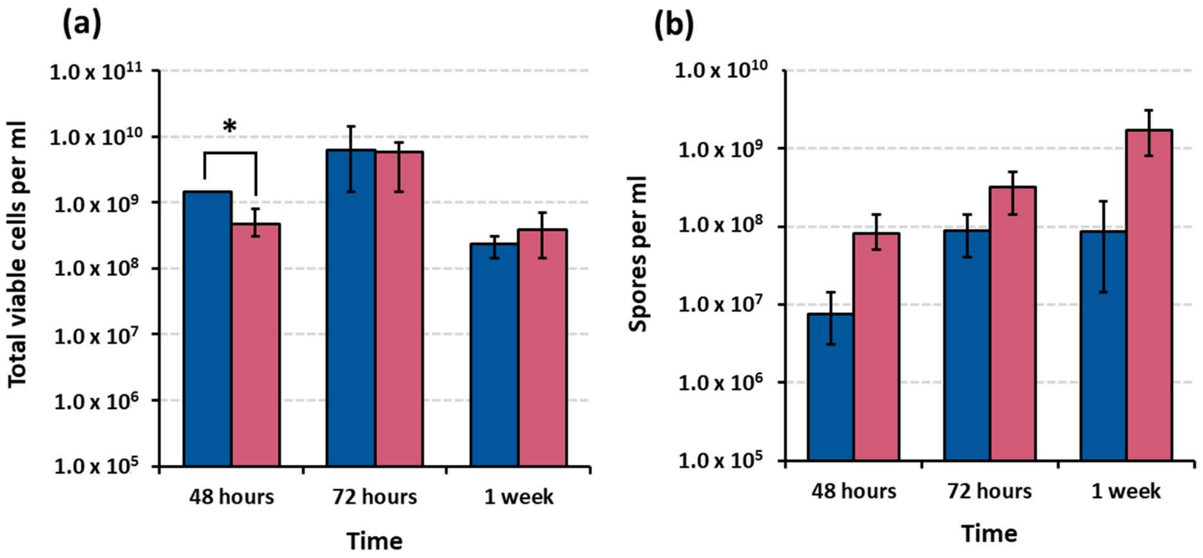

Figure S3. Viable cell and spore count of *C. botulinum* ST7B (blue) and V73 (pink) after 48 hours, 72 hours and 1 week based on MPN approach. Spore count was determined after heat treatment, as described in the Materials and Methods section. The experiment was performed in triplicates. Legend: * p-value < 0.05 with the t-test.

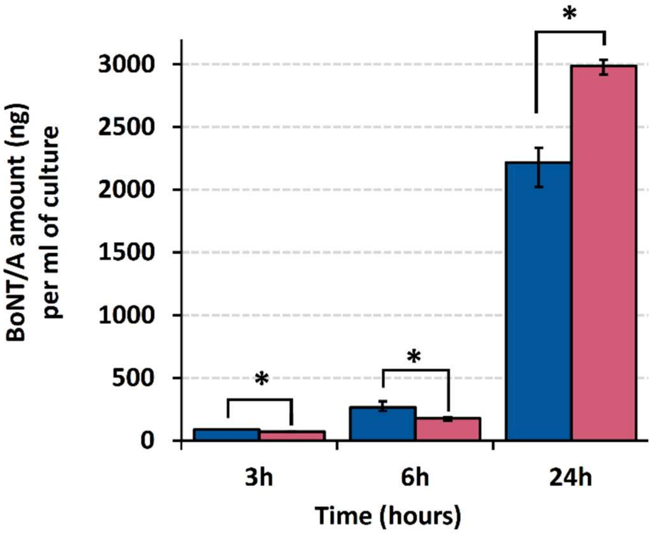

Figure S4. BoNT production of *C. botulinum* ST7B (blue) and V73 (pink) in TPGY medium. Error bars represent the minimum and maximum values among replicates. The experiment was performed in triplicate. Legend: * p-value < 0.05 with the t-test.

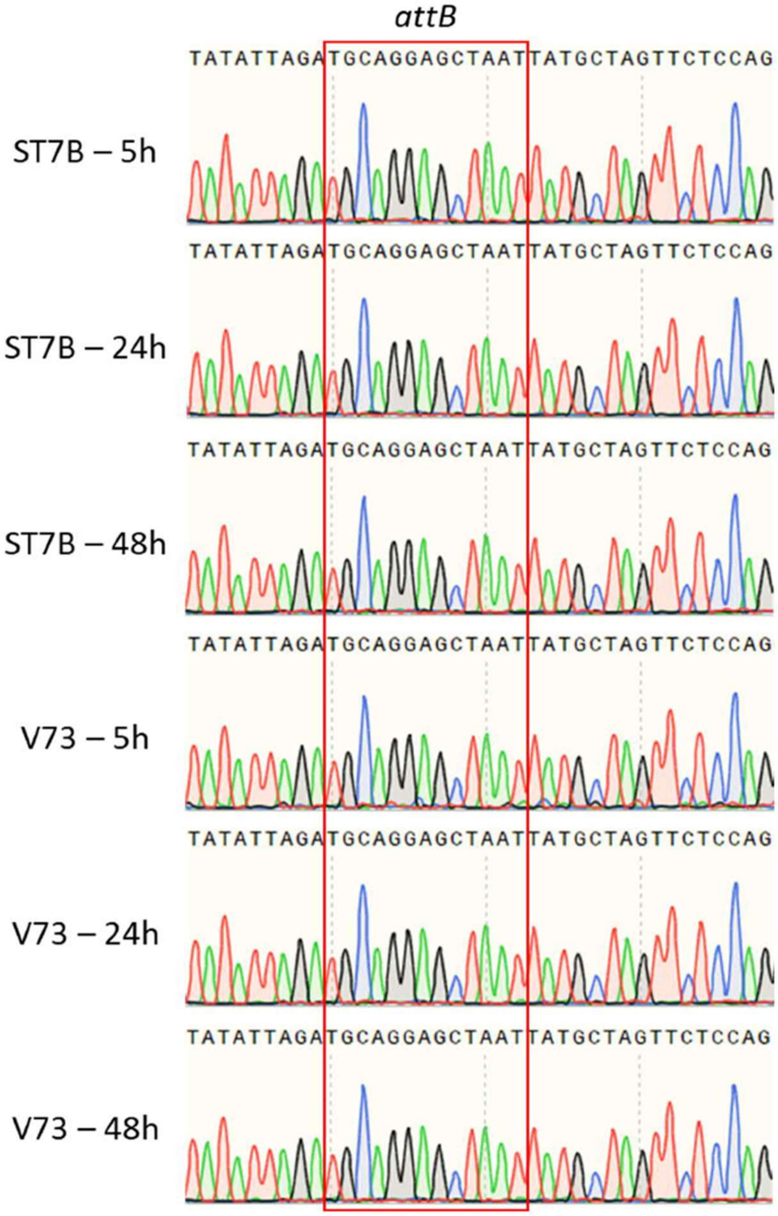

Figure S5. Sanger sequencing of the restored *yabG* gene in *C. botulinum* V73 at different time points. The *yabG* gene sequence of the ST7B counterpart was used a control.

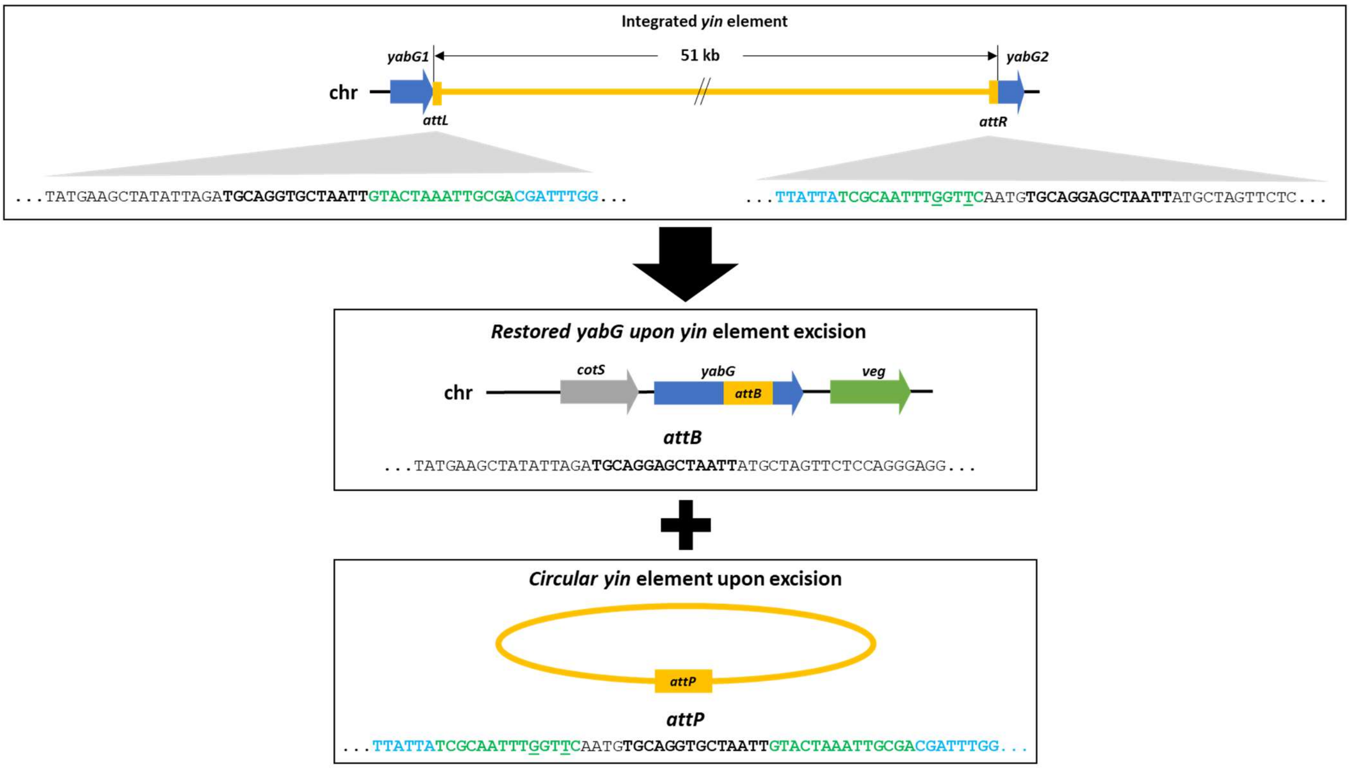

Figure S6. Identification of the attachment sites (*att*) based on the genome sequence of *C. botulinum* V73 and ST7B. The *attP* sequence was determined by Sanger sequencing (above). Legend: bold nucleotides, *attB* sites; blue and green nucleotides, asymmetric overlapping regions present in *attP*, *attL* and *attR*; underlined nucleotides, mismatched nucleotides.

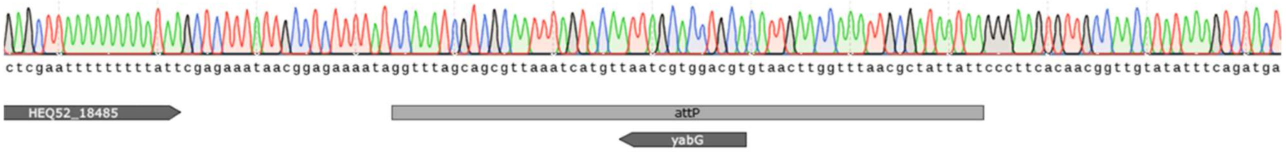

Figure S7. Sanger sequencing of the *attP* site present in the circular *yin* element in *C. botulinum* V73.

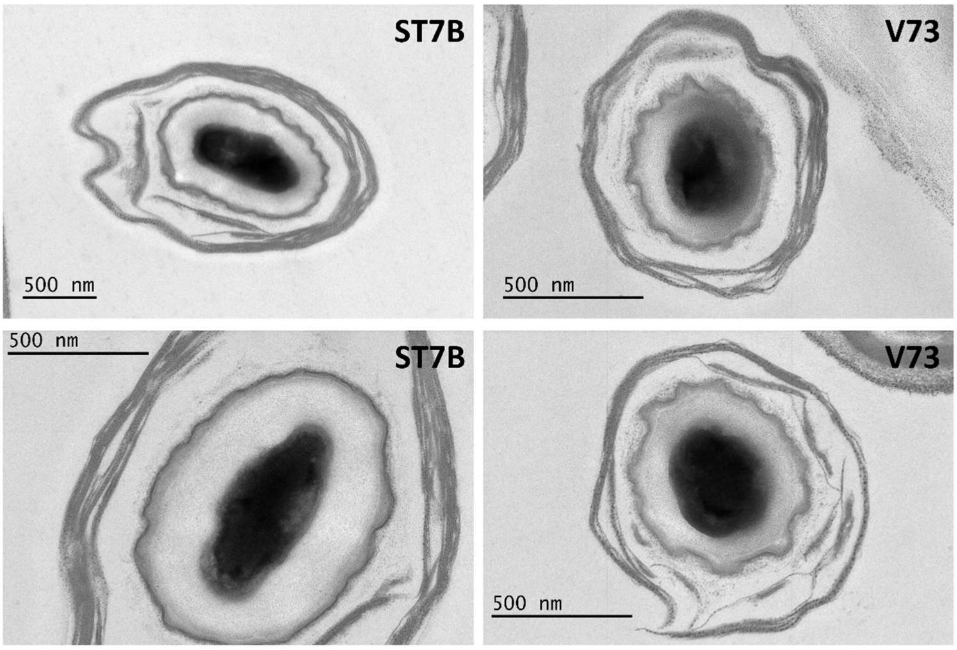

Figure S8. Transmission electron microscopy observations of individual *C. botulinum* ST7B and V73 spores.

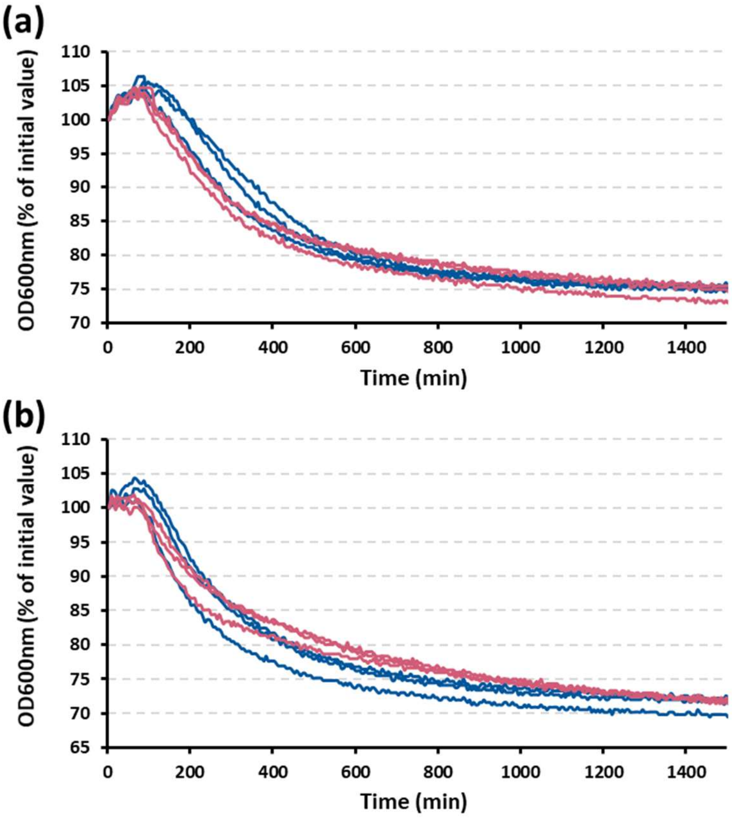

Figure S9. Spore germination assays of *C. botulinum* ST7B (blue) and V73 (pink). The germination buffer was either supplemented with L-alanine (**a**) or L-cysteine (**b**). For each condition tested, three technical replicates were measured.

Table S1. Primers used in the present study.

Primer name	Sequence (orientation 5′–3′)	Comments
yabG_rev	GGGGGATAAGGGAAAGCACT	Reverse primer located in *yabG*
yabG_for	CCACCAATTCCCTTTATACCTG	Forward primer paired with *yabG*_rev
yin_int_for	TCTTAGGCAGCGAACCAATT	Forward primer located in *yin*, paired with *yabG*_rev
circ_for	GTGACAGACGCAGAATGGAA	Forward primer located in *yin*, paired with *circ*_rev
circ_rev	GGTGGCTTAAATGGCAAGAT	Reverse primer located in *yin*, paired with *circ*_for
qPCR_yabG_for	GGCGCTTGTCAATCTTGCTA	Forward primer located in *yabG* (qPCR)
qPCR_yabG_rev	TTCACATAAAAATACTG-GATCTAAACA	Reverse primer paired with qPCR_*yabG*_rev
ctr_yabG_for	ACCATGACTAAGAAGGAAGAGGT	Forward primer located in *yabG*
ctr_yabG_rev	TGAAGAATTTTACCTGGTCTCCC	Reverse primer, paired with *ctr_yabG*_for

Table S2. List of genomic features predicted in the *yin* element.

Locus tag	Coordinate	Predicted gene product
HEQ52_18055	159...500	transcriptional regulator
HEQ52_18060	500...670	hypothetical protein
HEQ52_18065	667...3051 c	hypothetical protein
HEQ52_18070	3111...3413 c	hypothetical protein
HEQ52_18075	3426...3863 c	hypothetical protein
HEQ52_18080	4159...4323 c	hypothetical protein
HEQ52_18085	4444...4644 c	helix-turn-helix transcriptional regulator
HEQ52_18090	4692...5102 c	recombinase family protein
HEQ52_18095	5210...6109 c	SH3 domain-containing protein

HEQ52_18100	6251...6439 c	hypothetical protein
HEQ52_18105	6464...6742 c	hypothetical protein
HEQ52_18110	6795...7013 c	helix-turn-helix transcriptional regulator
HEQ52_18115	7224...7382 c	XkdX family protein
HEQ52_18120	7406...7573 c	hypothetical protein
HEQ52_18125	7577...9190 c	hypothetical protein
HEQ52_18130	9231 10289 c	RNA-dependent DNA polymerase
HEQ52_18135	10548...10892 c	diversity-generating retroelement protein Avd
HEQ52_18140	10977...11312 c	hypothetical protein
HEQ52_18145	11319...11723 c	hypothetical protein
HEQ52_18150	11726...12802c	YmfQ family protein
HEQ52_18155	12789...13862c	baseplate J/gp47 family protein
HEQ52_18160	13855...14286c	DUF2634 domain-containing protein
HEQ52_18165	14283...14534c	hypothetical protein
HEQ52_18170	14538...15530c	terminase
HEQ52_18175	15545...16162c	LysM peptidoglycan-binding domain-containing protein
HEQ52_18180	16178...18577c	hypothetical protein
HEQ52_18185	18624...19187c	hypothetical protein
HEQ52_18190	19209...19376c	hypothetical protein
HEQ52_18195	19409...19834c	XkdN-like protein
HEQ52_18200	19887...20315c	phage tail tube protein
HEQ52_18205	20333...21409c	phage tail sheath protein
HEQ52_18210	21413...21853c	hypothetical protein
HEQ52_18215	21856...22275c	HK97 gp10 family phage protein
HEQ52_18220	22276...22626c	hypothetical protein
HEQ52_18225	22628...22972c	phage head-tail connector protein
HEQ52_18230	23011...24033c	capsid protein
HEQ52_18235	24048...24374c	hypothetical protein
HEQ52_18240	24387...24980c	phage scaffold protein
HEQ52_18245	25000...25245c	hypothetical protein
HEQ52_18250	25443...25829c	ABC transporter ATPase
HEQ52_18255	25880...26218c	hypothetical protein
HEQ52_18260	26231...27250c	minor capsid proteinNT
HEQ52_18265	27243...28652c	phage portal proteinQAV
HEQ52_18270	28665...29921c	PBSX family phage terminase large subunit
HEQ52_18275	29896...30804c	phage portal protein
HEQ52_18280	30835...31029c	hypothetical protein
HEQ52_18285	31117...31425c	hypothetical protein
HEQ52_18290	31507...32739c	ParB N-terminal domain-containing protein
HEQ52_18295	32741...32947c	hypothetical protein
HEQ52_18300	33095...33670c	hypothetical protein
HEQ52_18305	33701...33931c	hypothetical protein
HEQ52_18310	33931...34227c	hypothetical protein
HEQ52_18315	34298...35038c	hypothetical protein
HEQ52_18320	35087...35287c	hypothetical protein
HEQ52_18325	35322...35585c	hypothetical protein
HEQ52_18330	35628...35822c	hypothetical protein
HEQ52_18335	35864...36010c	hypothetical protein
HEQ52_18340	36054...36245c	hypothetical protein
HEQ52_18345	36232...36444c	hypothetical protein
HEQ52_18350	36463...36609c	aspartyl-phosphate phosphatase Spo0E family protein
HEQ52_18355	36646...36807c	hypothetical protein

HEQ52_18360	36810...37262c	hypothetical protein
HEQ52_18365	37273...37479c	hypothetical protein
HEQ52_18370	37628...37792c	hypothetical protein
HEQ52_18375	37804...38109c	hypothetical protein
HEQ52_18380	38118...38444c	hypothetical protein
HEQ52_18385	38463...39218c	hypothetical protein
HEQ52_18390	39237...39470c	hypothetical protein
HEQ52_18395	39482...40003c	hypothetical protein
HEQ52_18400	40093...40575c	hypothetical protein
HEQ52_18405	40565...40933c	hypothetical protein
HEQ52_18410	40948...41508c	hypothetical protein
HEQ52_18415	41546...41707c	hypothetical protein
HEQ52_18420	41709...42053c	hypothetical protein
HEQ52_18425	42064...42885c	ATP-binding protein
HEQ52_18430	42857...43636c	DnaD domain protein
HEQ52_18435	43648...43929c	hypothetical protein
HEQ52_18440	44112...44309c	hypothetical protein
HEQ52_18445	44330...45196c	hypothetical protein
HEQ52_18450	45208...46230c	hypothetical protein
HEQ52_18455	46468...46695c	hypothetical protein
HEQ52_18460	46709...46870c	hypothetical protein
HEQ52_18465	46923...47084c	hypothetical protein
HEQ52_18470	47170...47352c	XRE family transcriptional regulator
HEQ52_18475	47594...48046	helix-turn-helix transcriptional regulator
HEQ52_18480	48082...48510	ImmA/IrrE family metallo-endopeptidase
HEQ52_18485	48746...50401	recombinase family protein

Table S3. Raw microscopy cell counts.

Time in hours	Strain	Replicate	Picture	Vegetative cells	Stage IV–V sporulating cells	Stage VI sporulating cells	Free phase bright spores	Free phase dark spores	Total
5 h	ST7B	1	1	253	0	0	0	0	253
		1	2	287	0	0	0	0	287
		2	1	517	0	0	0	1	518
		3	1	718	0	0	0	0	718
5 h	V73	1	1	568	0	0	0	0	568
		2	1	428	0	0	0	0	428
		3	2	319	0	0	0	0	319
24 h	ST7B	1	1	1192	0	0	0	0	1192
		2	1	673	0	0	0	0	673
		3	1	791	0	0	0	0	791
24 h	V73	1	1	530	296	29	1	0	856
		2	1	776	256	20	0	0	1052
		3	2	388	108	24	0	0	520
48 h	ST7B	1	2	224	1	4	0	0	229
		1	3	339	1	3	0	0	343
		2	2	430	2	2	0	0	434
		3	1	418	7	4	0	1	430
48 h	V73	1	1	117	8	163	1	0	289
		1	2	101	1	172	1	0	275
		2	1	92	5	202	0	0	299
		3	1	198	13	367	3	0	581
72 h	ST7B	1	4	281	2	16	0	0	299
		2	1	86	5	5	0	0	96
		2	2	156	1	4	0	0	161
		2	3	80	1	4	0	0	85
		3	1	76	0	6	0	0	82
		3	2	124	1	4	0	0	129
		3	3	123	1	4	0	0	128
72 h	V73	1	2	55	0	67	8	0	130
		1	3	42	2	51	9	0	104

		1	4	34	3	46	5	0	88
		2	2	26	0	77	8	0	111
		2	3	22	0	45	1	0	68
		2	4	23	0	53	3	0	79
		2	5	27	0	51	1	0	79
		3	1	15	4	56	5	0	80
		3	2	31	1	53	4	0	89
		3	3	26	0	54	4	0	84
		3	4	21	0	41	5	0	67
96 h	ST7B	1	1	92	1	2	0	0	95
		1	2	58	0	4	0	0	62
		1	3	57	0	2	0	0	59
		1	4	108	0	3	0	0	111
		2	1	81	0	4	1	2	88
		2	2	89	0	0	2	1	92
		2	3	120	0	1	0	0	121
		3	1	145	1	2	0	0	148
		3	2	120	0	5	0	0	125
		3	3	111	1	7	0	0	119
96 h	V73	1	2	18	1	49	63	0	131
		1	3	31	0	77	50	0	158
		1	4	29	0	52	15	0	96
		2	1	58	1	92	12	0	163
		2	2	40	0	92	21	0	153
		3	1	34	0	57	21	0	112
		3	2	83	0	0	13	1	97
		3	3	31	1	36	12	0	80
		3	4	24	1	35	10	0	70
120 h	ST7B	1	1	94	0	10	0	0	104
		1	2	106	2	1	0	0	109
		1	3	100	2	17	0	0	119
		2	1	44	2	9	0	0	55
		2	2	47	0	6	0	0	53
		2	3	45	1	2	1	0	49

		2	4	102	1	14	0	0	117
		2	5	175	6	28	0	0	209
		3	1	122	0	9	0	0	131
		3	2	115	0	15	0	1	131
		3	3	110	3	11	0	0	124
120 h	V73	1	1	43	0	80	48	0	171
		1	2	61	0	74	30	0	165
		2	1	54	0	108	41	0	203
		2	2	41	4	61	32	0	138
		3	1	31	1	54	38	0	124
		3	2	43	2	94	43	0	182
240 h	ST7B	1	1	103	5	36	16	5	165
		1	2	92	1	20	21	0	134
		2	1	94	0	8	9	0	111
		2	2	45	1	14	4	0	64
		2	3	83	1	17	11	0	112
		2	4	80	0	9	11	1	101
		3	1	87	2	24	9	0	122
		3	2	58	0	22	12	0	92
		3	5	67	2	15	12	1	97
240 h	V73	1	1	59	0	19	196	0	274
		1	2	54	1	9	258	0	322
		2	1	99	1	29	515	0	644
		3	1	94	1	26	410	4	535
